# Compliance to prescribing guidelines among public health care facilities in Namibia; findings and implications

**DOI:** 10.1007/s11096-020-01056-7

**Published:** 2020-05-26

**Authors:** Qamar Niaz, Brian Godman, Stephen Campbell, Dan Kibuule

**Affiliations:** 1 School of Pharmacy, Faculty of Health Sciences, University of Namibia, Windhoek, Namibia; 2 Division of Clinical Pharmacology, Department of Laboratory Medicine, Karolinska Institutet, Karolinska University Hospital Huddinge, 141 86 Stockholm, Sweden; 3 Strathclyde Institute of Pharmacy and Biomedical Sciences, University of Strathclyde, Glasgow, G4 0RE UK; 4 School of Pharmacy, Sefako Makgatho Health Sciences University, Pretoria, South Africa; 5 Centre for Primary Care, Division of Population Health, Health Services Research and Primary Care, University of Manchester, Manchester, M13 9PL UK; 6 NIHR Greater Manchester Primary Care Patient Safety Translational Research Centre, University of Manchester, Manchester, UK

**Keywords:** Compliance, Namibia, Prescribing indicators, Prescribing patterns, Qualitative research, Standard treatment guidelines

## Abstract

*Background* The World Health Organization estimates that over 50% medicines are prescribed inappropriately and the main driver of antimicrobial resistance globally. There have only been a limited number of studies evaluating prescribing patterns against national standard treatment guidelines (STGs) in sub-Saharan African countries including Namibia. This is important given the high prevalence of both infectious and non-infectious diseases in sub-Saharan Africa alongside limited resources. *Objective* Our aim was to assess prescribing practices and drivers of compliance to National guidelines among public health care facilities in Namibia to provide future guidance. *Setting* Three levels of public healthcare in Namibia. *Method* A mixed method approach including patient exit and prescriber interviews at three levels of health care in Namibia, i.e. hospital, health centre and clinic. *Main outcome measures* Medicine prescribing indicators, compliance to and attitudes towards National guidelines. *Results* Of the 1243 prescriptions analysed, 73% complied with the STGs and 69% had an antibiotic. Of the 3759 medicines (i.e. mean of 3.0 ± 1.1) prescribed, 64% were prescribed generically. The vast majority of prescribers were aware of, and had access to, the Namibian STGs (94.6%), with the majority reporting that the guidelines are easy to use and they regularly refer to them. The main drivers of compliance to guidelines were programmatic, that is access to up-to date objective guidelines, support systems for continued education on their use, and ease of referencing. Lack of systems to regulate noncompliance impacted on their use. *Conclusion* Whilst the findings were encouraging, ongoing concerns included limited prescribing of generic medicines and high use of antibiotics. A prescribing performance management system should be introduced to improve and monitor compliance to prescribing guidelines in public healthcare.

**Electronic supplementary material:**

The online version of this article (10.1007/s11096-020-01056-7) contains supplementary material, which is available to authorized users.

## Impact on practice

- There have only been a limited number of studies in sub-Saharan Africa evaluating compliance to standard treatment guidelines.
- This study shows that despite good access to standard treatment guidelines (STGs) by prescribers in Namibia, compliance to these guidelines is sub-optimal with high rates of antibiotic and brand name prescribing.
- Public healthcare systems in sub-Saharan Africa need to address programmatic barriers to enhance compliance to national guidelines
- Barriers to address to enhance the use of STGs include their design, the quality of evidence in the guidelines, the need for continued up-date guidance and education regarding their use, as well as systems to regularly audit prescribing practices.
- Pharmacists can play a key role in the development and dissemination of STGs including educating physicians on appropriate medicine use.

## Introduction

The appropriate use of medicines is critical especially in lower- and middle-income countries (LMICs) where the cost of medicines account for up to 70% of total healthcare expenditure, with potentially catastrophic implications for the family if a member becomes ill [1, 2]. The World Health Organization (WHO) estimates that over half of all medicines are inappropriately prescribed, dispensed or sold worldwide, and a similar percentage of patients fail to take their medicine properly [2].

In Namibia, several medicine use surveys have suggested the inappropriate use of medicines across all levels of health care [3, 4]. This is a concern as currently in Namibia over 45% of the adult population have hypertension [5], with cardiovascular diseases now a leading cause of death (21%) [5, 6]. There is also a high burden of infectious disease such as HIV/AIDS, tuberculosis, malaria and acute respiratory infections in Namibia [7–9]. In order to promote rational use of medicines (RUM), the Ministry of Health and Social Services (MoHSS) adopted the Essential Medicine concept with the first National Medicine Policy launched in 1998, and the first standard treatment guidelines (NSTGs) was launched in 1994, with a comprehensive update in 2012 [9]. STGs are seen as important interventions to improve medicine use in countries including Namibia [9–11]. However, compliance to the guidelines in 2014 was between 26.2 and 44.6% nationally [12], below the target of ≥ 90% with a rate of 80% considered acceptable [9].

### Aim of the study

The objective is to investigate current trends in prescribing practices and compliance with NSTGs among different level health care facilities in Namibia. In addition, qualitatively identify key factors that may influence prescribing practices and NSTG compliance.

### Ethics approval

Permission to conduct the research was granted by the University of Namibia (UNAM) and the Ministry of Health and Social Services (MoHSS, REF 17/3/3). Specific patient and prescriber identifiers and patient identifiers were not collected but rather codes were assigned to each study participant for identification.

## Methodology

### Study design and setting

A cross-sectional descriptive survey applying mixed methods was conducted to assess medicine prescribing patterns and drivers of compliance to NSTGs at three levels of health care in Namibia. These were the Intermediate Hospital Katutura (IHK), Katutura Health Centre (KHC) and Khomasdal Clinic (KMDC) in the Khomas Region. The Khomas region was chosen as it has a diverse cosmopolitan patient and prescriber population, a high population versus other regions in Namibia and concerns with adherence to STGs [12]. The Khomasdal clinic was purposely selected among the ten clinics in the region based on its proximity and similarity of demographics and services to IHK and KHC.

Quantitative methods were used to assess prescribing indicators based on those recommended by the WHO [9, 13]. Qualitative methods were applied to evaluate thematic drivers of compliance to NSTGs.

### Study population and sample

The target populations included outpatient prescriptions and prescribers at the three public health facilities. Prescriptions obtained from health passports during patient exit interviews were analysed for prescribing patterns. In Namibia, outpatient prescriptions are compiled in a medical booklet, the health passport. These detail consultation records including diagnoses, medical and medication history. This study only included prescribing data on recent prescriptions at the three facilities. Consequently, a sample of 584 patient prescriptions was determined using Kish and Leslie [14] method for a single sample estimation of proportion [9, 14]. However, since the study was conducted at two different levels of health care, we estimated the total sample at 2 * 584 = 1168. We included an additional 6.5% to account for prescriptions that may have missing data. As a result, a maximum number of prescription records to be collected at patient exit interviews was 1243. These prescriptions were collected from 1243 patients who were sampled from daily outpatient registers at the respective outpatient pharmacies at the three health facilities. A systematic sampling method, i.e. every third patient, was used to consecutively recruit patients. Of the 7 (0.56%) prescriptions with presenting complaints but no diagnosis indicated, 5 were rectified after consultation with the respective prescribers and the two were replaced through the process of systematic sampling matched with the age and sex of the patients.

Each prescription included information on patient demographics, diagnosis, medication and prescriber. Prescribers (both nurses or doctors) are required by law to indicate the diagnosis on the prescriptions as classified in the NSTGs. For prescriptions without a clear diagnosis, a team consisting of a pharmacist, doctor and nurse reviewed the prescriptions to link the diagnosis with the disease categories in the NSTG.

Secondly, prescribers who were on duty during the 6 months study period, 1st February to 31 July 2015, were interviewed for drivers for compliance to NSTGs. The sample of prescribers was determined purposively. A total of 74 prescribers working at the three public health facilities at the time of data collection were included. Of these, 44 were employed at IHK, 21 at KHC and 9 at KMDC. At any given time, 12 prescribers worked at OPD in IHK, 8 at KHC and 5 at KMDC.

We used the duty rosters to identify prescribers working at out-patient departments in the selected three sites to include them in the sample. Using the roster, 40 prescribers were expected to work in three sites at OPD during the data collection period. All 40 prescribers were selected for prescriber interviews. We did not include any prescribers in the inpatient department of IHK or any working in specialised clinics. Responses were broken down by prescriber type for further analysis.

### Data collection procedure

Data were collected in two phases; patient exit interviews on medicine prescribing patterns and prescriber interviews using a structured questionnaire for drivers of compliance to NSTGs.

### Patient exit-interviews

Patients were recruited into the study using a systematic sampling technique, i.e. every third patient registered at the outpatient’s pharmacy.

Only patients that gave written informed consent were subsequently interviewed and prescriptions analysed. The 1243 patients/patients were stratified by health facility, with the allocation calculated on the basis of patient turnover resulting in KMDC (10%), KHC (35%) and IHK (55%). Only prescriptions from the general outpatient department were selected. Prescribing data were abstracted from patients’ prescription booklets (i.e. health passports) by the researchers (QN and DK) and a team of three experienced data collectors using the WHO recommended tool for medicine use evaluation [13]. We also excluded prescriptions with incomplete information such as missing diagnosis or missing details of the patient. Two patients’ prescriptions had missing data on diagnosis. Data were quantitatively analysed to determine the prescribing indicators [9].

### Survey of prescribers

A questionnaire (Annex 1) was administered to prescribers at the selected health facilities to assess for drivers of compliance to NSTGs. The tool was piloted with the help of two intern doctors at IHK and standardized before being rolled out. Prescribers’ details were also collected.

Data to determine the level of compliance and the use of NSTGs in the prescribing of medicines was collected using a self-administered questionnaire. Only prescribers whose names appeared on the prescriptions evaluated in the first phase of the study were assessed. During the collection of the questionnaire, a structured interview was conducted with the prescribers to assess the availability and access to NSTGs as well as gain further insight on key factors that might impact on their prescribing practices and compliance to NSTGs. The interviews were structured in such a way that the answers could be thematically analysed and/or quantified for ease of analysis. All interviewees gave their informed consent before being interviewed.

### Data analysis

The main outcome measures were medicine prescribing practices and qualitative determinants of compliance to NSTGs among public health care facilities in Namibia. Quantitative data from the patient exit and prescriber interviews were entered into Epidata 3.1 for management and exported to SPSS v24 for descriptive analysis of the indicators and compliance to NSTGs.

Prescribing practices were analysed using descriptive statistics as per the WHO/INRUD indicators [9, 13]. The five indicators and the MoHSS targets include:

- Average number of medicines per out-patient prescription
- The percentage of medicines prescribed by generic name
- Percentage of prescriptions with an antibiotic
- Percentage of prescriptions with an injection
- The level of compliance to STGs

Quantitative and qualitative methods were applied to identify the drivers of compliance of NSTGs. Descriptive statistical analysis was used to determine the level of awareness, availability, access, use, and training on NSTGs. The drivers of compliance to STGs were also quantitatively determined using the χ^2^ test with the level of significance (α) set at *p* = 0.05 and a 95% confidence interval, with qualitative data analysis conducted using thematic content analysis to identify the themes and subthemes of drivers of compliance to the NSTGs. Thematic content analysis was performed manually from data obtained from the interviews. The content or responses to the question items were colour coded and organized into sub-themes. The significant drivers of compliance to NSTGs were subsequently converged during the analysis to support the themes.

## Results

### Demographics

A total of 37 prescribers were interviewed giving a response rate of 92.5% (37/40). Table 1 shows that the majority of the prescribers interviewed were from the hospital and were medical officers.

**Table 1:** Distribution of prescribers by professional cadre and health facility

Demographic	Prescriber cadre		Total	χ^2^	*P* value	Cramer V
	Medical	Nursing				
*Facility level*						
Hospital	23	2	25	14.15	0.000*	0.681
PHC	4	8	12			
*Health facility*						
IHK	23	2	25	19.56	0.000*	0.727
KHC	2	8	10			
KMDC	2	–	2			
*Cadre*						
Enrolled nurse	–	4	4	37	0.000*	1
Medical intern	3	–	3			
Medical officer	20	–	20			
Registered nurse	–	8	8			
Student nurse	–	2	2			
*Sources of information*						
Algorithm charts	0	2	2	17.7	0.013*	0.692
Ward protocols	1	–	1			
Formularies	6	–	6			
Leaflets	–	1	1			
Online resources	3	–	3			
Medical textbooks	2	1	3			
Treatment guidelines	4	5	9			
No response	11	1	12			

*(*p* < 0.05)-statistically significant—Pearson χ^2^ test

### Medicine prescribing practices

#### Compliance with NSTGs

The average number of medicines per prescription, the percentage prescribed by their generic/INN (International non-proprietary) name and the percentage including an antibiotic are contained in Table 2. Table 2 also contains government targets. There was no statistical difference between the various healthcare levels. Injections were prescribed in 10.8% of prescriptions, highest in hospital outpatients [9].

**Table 2:** Compliance with WHO INRUD Criteria including Namibian standard treatment guidelines. Adapted from [9, 15]

WHO/INRUD indicator	WHO targets	Namibia ministry of health targets		Indicator measures
		Target	Acceptable	
Average number of medicines per prescription	< 2	< 2	2.5	3.0 ± 1.1
% of prescriptions with an antibiotic	< 30%	< 25%	35%	69%
% of prescriptions with an injection	< 20%	< 10%	15%	10.8%
% of medicines with generic name	100%	100%	80%	64%
Compliance to NSTG	> 80%		> 80%	73%

Out of the 1243 prescriptions, the majority complied with NSTG recommendations (Table 2), with compliance significantly higher among PHC facilities [76.1% (n = 416/547)] than the hospital [70.5% (n = 491/696, *p *= 0.03)].

#### Awareness and utility of NSTGs

**Fig. 1:**
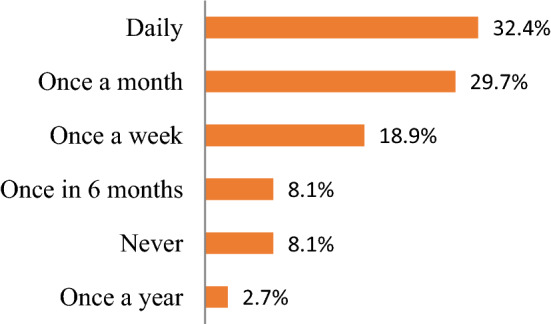
Frequency of use of the STGs (n = 37)

The majority of the prescribers (94.6%) were aware and had access to the NSTGs for reference purposes (Table 3), with 82% reporting that it is easy to use the NSTGs. 32.4% reported that they refer to NSTGs on a daily basis with 18.9% once a week (Fig. 1). However, only 18.9% of prescribers had received at least one training session on the use of the STGs (Table 3).

**Table 3:** Distribution of awareness and use of STGs by prescribers’ cadre

Demographic	Prescriber cadre		Total	χ^2^	*P* value	Cramer V
	Medical	Nursing				
*Awareness of STG*						
Yes	25	10	35	0.783	0.376	0.145
No	2	–	2			
*Access to STG copy*						
Yes	26	9	35	0.566	0.452	0.124
No	1	1	2			
*Training on STG use*						
Yes	5	2	7	0.01	0.919	0.017
No	22	8	30			
*Frequency of STG use*						
Daily	7	5	12	3.905	0.563	0.325
Never	3	–	3			
Once a month	8	3	11			
Once a week	5	2	7			
Once a year	1	–	1			
Once in 6 months	3	–	3			
*Ease of STG use*						
Difficult	4	2	6	1.266	0.531	0.185
Easy	20	8	28			
*Advantages of STG use*						
Comprehensive	5	–	5	3.963	0.139	0.327
Easy indexing	10	7	17			
No response	12	3	15			

*(*p* < 0.05)-Statistically significant—Pearson χ^2^ test

#### Sources of information

Prescribers used a wide variety of references sources when prescribing medicines, ranging from patient leaflets to local and international STGs and/or treatment protocols.

**Fig. 2:**
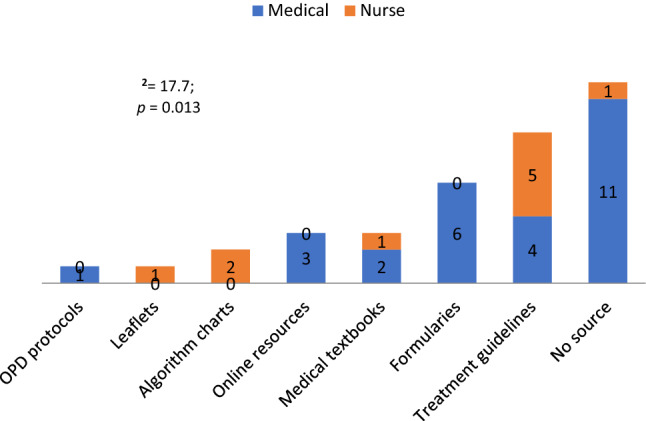
Sources of information used when prescribing medicines (n = 37)

48% used printed guidelines in the form of STGs, formularies and algorithm charts when seeking sources of prescribing information (Fig. 2). The most used printed treatment guidelines were the NSTG; disease specific guidelines for HIV/AIDS, antiretroviral therapy, tuberculosis, malaria, and sexual transmitted infections; the South African guidelines; the PHC manual; as well as flow charts and treatment protocols. Online sources of information included Medscape and Wikipedia.

### Main thematic drivers for compliance to NSTGs

The majority of the prescribers reported the simple indexing layout of the NSTGs, their access and tailored information, as the main factors driving their use (Table 4).

**Table 4:** Factors promoting the use of STGs

Demographic	Prescriber cadre		Total	χ^2^	P value	Cramer V
	Medical	Nursing				
*Pros for STG use*						
Comprehensive	5	–	5	3.963	0.139	0.327
Easy indexing	10	7	17			
No response	12	3	15			
*Cons for STG use*						
Access to STGs	2	4	6	11.346	0.078	0.554
Access to medicines	2	–	2			
Information overload	2	3	5			
Out-dated; needs review	3	–	3			
Does not fit in pocket	2	–	2			
No response	16	3	19			

*(*p* < 0.05)-Statistically significant—Pearson χ^2^ test

The principal factors included:

- **Comprehensiveness of the STG:** 5 responses supported the statement that the STGs cover the treatment and pathogenesis of common disease conditions at all levels of health care in Namibia. Some of the responses included; “Common diseases are found here, the treatment and pathologic conditions are clear” and “Practical, straightforward and inclusion of most conditions”.
- **Simple indexing system and easy to understand:** 17 responses confirmed the NSTGs are well laid out to facilitate quick identification of disease conditions and medicines. Two responses though were against the layout of the NSTG due to lack of time to make quick references (Table 3). Such responses included: “lack of time to page through the STG with high workloads” and “It is a bit difficult because you have to have enough time to sit and page back in the book and prescribe from it”. Against this, 83% of responders agreed the NSTGs are simple, clear and easy to understand, have a good lay out and good use of a colour coding system. Some of the responses included: “Clear and easy to understand”, “each condition in STGs is clearly set out”, “They are easy to use because the index is clearly stating according to the alphabetical order plus the medicine index on conditions are separate which makes it even easier to use.” “Pages are coloured coded and the STG is easy to carry”. “Treatment is clearly laid out step by step; well compiled”.
- **Access/availability of STGs by health workers:** All three responses on this theme reported a lack of access and availability of NSTG copies: “We have to share the book so sometimes you have to wait for it if your colleagues are busy with it”. “They are not easily available”.
- **Availability of recommended STG medicines at the facility:** One response indicated the lack of certain medicines recommended by the STG demotivated them from using the STG: “Some medications are not in stock that is supposed to be used according to the STG”. However, two responses reported that the medicines found in the STGs are available.
- **Relevance of information to health care cadre or health facility level:** Two prescribers indicated that the information in the STG is not structured for application by different prescribers - certain aspects are irrelevant or challenging to understand. Responses included: “Information is not clear on how to give the treatment and there are no second choices in case if the medicines are not available”.
- **Portability of the STG:** Concerns (2 responses) included; “Not pocket fit. It is too thick, difficult to carry around”
- **Updated or objective information:** There was one response that the information in the STG is not up-to-date as well as trusting the evidence in the STGs: “Not evidenced based”

#### Remedial strategies for effective use of NSTGs in prescribing of medicines

The prescribers suggested a number of interventions to improve compliance which are contained in Table 5.

**Table 5:** Strategies to increase the use of STGs

Strategy (theme)	Suggestions for the future
Access to essential medicines	The medicines included in the essential medicine list (Nemlist) should be available all the times. The medications listed for treatment in the STG should be on the Nemlist and available in stock always
Training on use of STG/refresher courses	The MoHSS should provide continuous refresher courses for prescribers; this will promote prescribers to make correct references of symptoms and treatments “There is a need to include a list of available medicine in each health facility level as well as their common side effects”
Updating guidelines	Prescribers recommended posting of guidelines for identified recurrent problems (wrong prescriptions). Make them more available; update them to match current global medical guidelines. “The STGs should be up-to-date and based on current literature”. “It needs to be updated to accommodate the hospital level fully (and not referred to the hospital)”
Access and availability of STG	Every staff member must have his/her own book. STGs should be available commercially at reasonable price. STGs should be available more frequently. “STG should be available at all health facilities, wards and out-patient units”
Organization of the STG	Make the smaller and more specific. “Make the STGs more focussed on nursing diagnosis and not general diagnosis”. “STGs must be revised and written according to the health workers’ category e.g. Management for nurses and doctors”. “Too much information for one condition it required a lot of time”. Direction on interpreting the main signs and symptoms: “Please note first the sign and symptoms of different diseases than the diagnosis and the treatment. “Clearly outline 1st option for prescribing and Second option for prescribing in case if the patient comes back with the same problem”. Reduce the size of the STG and make it pocket fit: “STGs should be short and concise”
STG audits	Conduct regular evaluation on the use of the STG to make sure health workers adhere to it

## Discussion

We believe this is the first study to qualitatively identify key factors influencing prescribing practices and NSTG compliance across disease areas building on assessments of guideline adherence in specific treatment and disease areas as well as ways to improve the content and pragmatism of national STGs among sub-Saharan African countries [10, 16–18]. This is a concern given the high prevalence of both infectious and non-infectious diseases in sub-Saharan Africa and their impact on morbidity, mortality and costs [5, 19–27].

The prescribing indicators were typically sub-optimal compared with the Namibia and WHO/INRUD standards, similar to other African countries [9, 15]. However, 73% of prescriptions were compliant to NSTG recommendations, an improvement on previous studies in Namibia [12], comparing favourably with recent studies among PHCs in Botswana and other LMICs [28–31]. However, lower than the compliance level set at 85% for Namibia [9], with ongoing concerns that antibiotic prescribing remains suboptimal [9].

Encouragingly, there was a high level of awareness and availability (94.6%) of the NSTGs among prescribers (Table 2), similar to the previous study by Akpabio et al. in Namibia and Matsitse et al. in South Africa [12, 31]. This compares with variable availability of STGs among PHCs in Botswana [32]. However, 8.1% of prescribers in Namibia had never seen a copy of the NSTG (Fig. 1) and never made reference to the NSTG in their prescribing. Encouragingly as well, 32.4% of prescribers routinely referred to the NSTG on a daily basis when making prescribing decisions with 18.9% referring the NSTG once a week, higher than the previous study by Akpabio et al. [12]. In addition, a high number of prescribers (82%) found it easy to refer to the NSTGs when needed although concerns with the lack of training (Table 2) similar to South Africa [31].

The prescribers reported using a wide variety of reference sources when prescribing medicines (Table 1, Fig. 2). Most prescribers used printed guidelines in the form of STGs, as well as treatment and algorithms charts (Fig. 2), similar to Uganda [33]. Encouragingly, there was no mention of pharmaceutical companies as a source of information different to some LMICS [34–38], with the potential for biased information affecting subsequent prescribing and patient care [38–41].

The high use of the STGs appeared to be due to a number of factors including their comprehensiveness, simple and well-structured STGs, availability, relevance, objectivity and portability (Table 3). Training on STGs has reduced the prescribing of antibiotics and over use of injections in other countries [42, 43]. Objectivity and trust in prescribing guidance resulted in high adherence rates to the ‘Wise List’ in Stockholm County Council in Sweden [44–46].

Recommendations on drivers for compliance to NSTGs (Table 4) included increasing access to STGs and essential medicines at health facilities, continuous professional training, regularly updating, and continuously auditing and monitoring prescribing against NSTGs. These findings are also in line with a similar previous study conducted in a number of regions of Namibia [12].

### Limitations

We are aware of a number of limitations with this study. The principal limitation is that the study was carried out in only one region of Namibia and with a limited number of health facilities. However, we believe our findings are robust based on the nature of the chosen sites and their representational characteristics. As a result, providing future guidance on ways to improve medicine use throughout Namibia and wider.

## Conclusion

Whilst the overall awareness of STGs is high among prescribers in Namibia, their use can be limited. The main factors driving the use of the STGs in Namibia are their access, the availability of medicines recommended by the STG, the simplicity and objectivity.

The findings suggest that STGs should be regularly revised, routinely made available to all health professionals, and the treatment options described in STGs should be available and in stock at all times. Pharmacists can also play a key role here. These are considerations for the future along with the introduction of a prescribing performance management system including agreed quality indicators. Pharmacists can play a key role in their development. The introduction of electronic prescribing systems can help with real time auditing of prescribing as seen with the Wise List in Sweden [46, 47].

## Electronic supplementary material

Below is the link to the electronic supplementary material.

Supplementary material 1 (DOCX 16 kb)
