# Supplementary material for: Compliance to prescribing guidelines among public health care facilities in Namibia; findings and implications
Source: Int J Clin Pharm. 2020 May 26;42(4):1227–36. doi: 10.1007/s11096-020-01056-7 (PMC7476965; doi:10.1007/s11096-020-01056-7)
Supplement: Supplementary file 1 — Supplementary material 1 (DOCX 16 kb) [file 11096_2020_1056_MOESM1_ESM.docx]

**Supplementary Material (Table A1) – Questionnaire**

**Thank you for accepting to take part in this assessment**

***Question 1:***

Are you aware of the comprehensive Namibia Standard Treatment Guidelines (STGs)?

(1)□ Yes (2) □ No

***Question 2:***

Do you have STGs available in your work place?

(1)□ Yes (2) □ No

If Yes, Please show me the copy of STGs.

***Question 3:***

Have you received training on the use of STGs?

(1)□ Yes (2) □ No

**Question 4:**

How often do you use STGs while prescribing treatment for your patients?

(1)□ Daily (2) □ Once in a week (3) □Once in a month

(4) □ Once in 6 months (5) □ Once in a year

**Question 5:**

How do you find the use of STGs in your daily practice?

(

1)□ Easy (2) □ Difficult

**Question 6:**

Please list the reasons which make the use of STGs easy or difficult for you depending upon your answer of the Question 5?

_______________________________________________________________________________________________________________________________________________________________________________________________________________________________________________________________________________________________________________________________________________________________________________________

**Question 7:**

What are your recommendations to overcome difficulties in the use of STGs?

_______________________________________________________________________________________________________________________________________________________________________________________________________________________________________________________________________________________________________________________________________________________________________________________

**Question 8:**

List the sources of information that you use in your practice in addition to STGs while choosing treatment for your patients?

___________________________________________________________________________________________________________________________________________________________________________________________________________________________________________________________________________________________________________________________________________________________________

***Thank you for accepting to take part in this assessment***
